# Using linear parameter varying autoregressive models to measure cross frequency couplings in EEG signals

**DOI:** 10.3389/fnhum.2022.915815

**Published:** 2022-09-16

**Authors:** Kyriaki Kostoglou, Gernot R. Müller-Putz

**Affiliations:** ^1^Institute of Neural Engineering, Graz University of Technology, Graz, Austria; ^2^BioTechMed-Graz, Graz, Austria

**Keywords:** cross frequency coupling (CFC), linear parameter varying (LPV), autoregressive (AR), electroencephalogram (EEG), spinal cord injury (SCI)

## Abstract

For years now, phase-amplitude cross frequency coupling (CFC) has been observed across multiple brain regions under different physiological and pathological conditions. It has been suggested that CFC serves as a mechanism that facilitates communication and information transfer between local and spatially separated neuronal populations. In non-invasive brain computer interfaces (BCI), CFC has not been thoroughly explored. In this work, we propose a CFC estimation method based on Linear Parameter Varying Autoregressive (LPV-AR) models and we assess its performance using both synthetic data and electroencephalographic (EEG) data recorded during attempted arm/hand movements of spinal cord injured (SCI) participants. Our results corroborate the potentiality of CFC as a feature for movement attempt decoding and provide evidence of the superiority of our proposed CFC estimation approach compared to other commonly used techniques.

## Introduction

Interactions of brain oscillations in different frequency bands, also known as cross frequency couplings (CFC), have been proposed to serve as a mechanism for neural coordination across different spatiotemporal scales (Canolty and Knight, 2010). The most common form of CFC is phase-amplitude coupling, whereby the phase of the lower frequency rhythms modulates the amplitude of spatially localized high frequency oscillations. This type of modulation allows for efficient control of communication and information transfer between brain regions. In humans, phase-amplitude CFC has been observed in multiple brain areas based on recordings obtained at different levels, i.e., from intracellular to surface electroencephalogram (EEG) measurements and under different experimental conditions (Mormann et al., 2005; Canolty et al., 2006; Cohen et al., 2008; De Lange et al., 2008; Penny et al., 2008; Tort et al., 2009; Axmacher et al., 2010; Seeber et al., 2014; Gwon and Ahn, 2021) or pathological conditions (López-Azcárate et al., 2010; Özkurt and Schnitzler, 2011; De Hemptinne et al., 2013; Edakawa et al., 2016; Combrisson et al., 2017; Wang et al., 2017).

In non-invasive brain computer interfaces (BCI), the concept of CFC has not yet been fully explored. To date, only a few studies have investigated the prospect of CFC in BCI systems (Gysels and Celka, 2004; Wei et al., 2007; Dimitriadis and Marimpis, 2018; Georgiadis et al., 2019; Feng et al., 2020; Gwon and Ahn, 2021). Gysels and Celka (2004) and Wei et al. (2007) used CFC as a measure of functional connectivity between different brain regions and demonstrated its potentiality as a feature for mental task classification. In Dimitriadis and Marimpis (2018), CFC was estimated within EEG sensors from single trials during experimental paradigms that generated visually evoked potentials. By employing CFC as a feature for decoding flashing images and stimulus presentations, high classification accuracy and information transfer rates were achieved. Similarly, in Georgiadis et al. (2019), Feng et al. (2020), and Gwon and Ahn (2021) CFC was introduced as the main building block of motor imagery based decoding schemes.

When it comes to CFC estimation, there is no golden rule that applies in selecting an appropriate method. Some of the most commonly used CFC estimation techniques are the Phase-locking Value (PLV) (Lachaux et al., 1999; Cohen, 2008), the Mean Vector Length (MVL) (Canolty et al., 2006), the Kullback–Liebler based Modulation Index (MI) (Tort et al., 2010), and the Generalized Linear Model (GLM) (Penny et al., 2008). Each method was developed based on different principles and exhibits unique advantages but also limitations. The PLV compares the phase of the instantaneous high frequency amplitude with the low frequency phase. The MVL computes the magnitude of the average complex signal defined by the instantaneous high frequency amplitude and the instantaneous low frequency phase. The MI measures the deviation of the distribution of the high frequency amplitude, with respect to the phase of the low frequency, from the uniform distribution and the GLM is simply a linear regression model fitted on the instantaneous high frequency amplitude using the cosine and the sine of the instantaneous low frequency phase as regressors.

Herein, we provide an alternative CFC estimation method based on Linear Parameter Varying Autoregressive (LPV-AR) models (Tóth, 2010). CFC is a non-linear phenomenon and therefore requires non-linear methods for its estimation. In contrast to the simple linear AR models, LPV-AR models are able to capture non-linear dynamics and interactions. The unique characteristic of these models is that their coefficients can be modulated by an external variable/signal and, more importantly, they can be identified using the Least Squares approach since the LPV-AR is linear in the parameters. The main idea is to use the instantaneous low frequency phase as the external modulating signal of an AR model fitted on the instantaneous high frequency amplitude. AR models have been widely employed for power spectrum estimation. Specifically, the AR coefficients can be used to describe the power spectrum of a signal. By allowing an external variable to modulate these coefficients, we are modeling the time-varying spectral changes of the signal under consideration induced by the external variable. There has been one previous study on LPV-AR based CFC (la Tour et al., 2017), however, their approach in estimating CFC is completely different than the one that we describe here. The model of La Tour et al. is applied directly on the raw signals, in contrast to the instantaneous amplitude and phase as usually done in CFC estimation. However, filtering and preprocessing is required in order to isolate the low-frequency driver and remove its effects from the raw signal. The model also assumes time-varying model innovations which requires the use of non-linear optimization techniques for model estimation. On the other hand, our proposed approach provides closed-form solutions to a linear Least Squares problem.

Our proposed LPV-AR based CFC methodology was validated using both synthetic and real data. The real data comprised of EEG recordings obtained during hand/arm movement attempts of spinal cord injured (SCI) participants. Our main goal is to encourage the use of CFC as a decoding feature in future BCI applications but also validate the capabilities of our proposed LPV-AR based CFC measure using real EEG recordings.

## Materials and methods

### Linear parameter varying autoregressive model based cross frequency coupling

In this section we describe the LPV-AR modeling technique and its application on CFC estimation.

#### Linear parameter varying autoregressive model

In an autoregressive (AR) model, the signal of interest is expressed as a linear combination of its past values (Ljung, 1998),


(1) $$y\,\left(n\right)=\sum_{k=1}^{p}a_{k}\,y\,\left(n-k\right)+\varepsilon \,\left(n\right)$$

where *n* represents the discrete time, ***y*** ∈ ***R**^N×1^* is the observed signal, *N* is the total number of samples, *p* is the model order (i.e., the number of past time lags that are taken into account), *a*_*k*_ are the AR coefficients for each order *k* and ε ∈ ***R**^N×1^* is a zero-mean white noise vector. In LPV-AR systems, the coefficients *a*_*k*_ are modulated by a time-varying external signal ***s*** ∈ ***R**^N×1^* known also as scheduling variable. Eq. 1 is then expressed as (Tóth, 2010),


(2) $$y\,\left(n\right)=\sum_{k=1}^{p}a_{k}\,\left(s_{n}\right)\,y\,\left(n-k\right)+\varepsilon \,\left(n\right)$$

where for notational simplicity *s*_*n*_ = *s*(*n*). Note here that *a*_*k*_ has a static dependence on ***s*** (i.e., it only depends on the instantaneous values of ***s*** at each time point *n*). Identification of the LPV-AR model of Eq. 2 can be addressed by recasting the problem as linear regression. Specifically, the AR model coefficients {*a*_*k*_} are expressed as a linear combination of a set of known fixed basis functions,


(3) $$a_{k}\left(s_{n}\right)=\theta_{k}^{\text{T}}\Psi_{k}\left(s_{n}\right)=\theta_{k_{0}}+\sum_{i=1}^{q}\theta_{k_{i}}\psi_{k_{i}}\left(s_{n}\right),k=1:p$$

where *q* is the number of basis functions. Herein, we selected a polynomial basis due to its simplicity. Compared to other basis functions (e.g., radial basis functions) that require tuning of multiple hyperparameters, the polynomial basis requires only the selection of a polynomial order *q*, e.g., $\Psi_{k}^{T}\,\left(s_{n}\right)=\left[1,s_{n},\ldots ,s_{n}^{q}\right]$. Furthermore, polynomial functions are widely used in biosignal processing and physiological systems (Mitsis and Marmarelis, 2002; Marmarelis, 2004; Zhang et al., 2010). Based on the polynomial basis, Eq. 2 can be expressed as,


(4) $$y\,\left(n\right)={\text{w}}^{T}\,\varphi \,\left(n\right)+\varepsilon \,\left(n\right)$$

where,


(5) $$\text{w}={\left[\theta_{1_{0}},\theta_{1_{1}},\ldots \,\theta_{1_{q}},\ldots ,\theta_{p_{0}},\ldots \,\theta_{p_{q}}\right]}^{T}\in {\text{R}}^{D\times 1}$$

with *D* being the total number of coefficients i.e., *D* = *p*(1 + *q*) and φ(*n*) is the extended regressor,


$$\varphi \left(n\right)=[y\left(n-1\right),s_{n}y\left(n-1\right),\ldots s_{n}^{q}y\left(n-1\right),\ldots ,$$

(6) $$y\left(n-p\right),s_{n}y\left(n-p\right)\ldots ,s_{n}^{q}y\left(n-p\right)]{}^{T}$$

For each order *p* one could define a different *q* value, however, for simplicity we kept *q* fixed. If *N* is the total number of samples (i.e., *n* = 1…*N*), the data can be reorganized in the following form,


(7) $$\Phi ={\left[\varphi \,\left(1\right),\ldots \,\varphi \,\left(N\right)\right]}^{T}\in {\text{R}}^{N\times D}$$

In matrix form, Eq. 4 can be written as,


(8) y=Φ⁢w+ε

where ***y***,ε ∈ ***R**^N×1^*. The unknown coefficients **w** can be estimated using the Least Squares (LS) solution,


(9) $${\text{w}}_{\mathrm{LS}}={\left(\Phi^{T}\,\Phi \right)}^{-1}\,\Phi^{T}\,y$$

In addition, if we apply ridge regression, the regularized LS (RLS) solution is given as,


(10) $${\text{w}}_{\mathrm{RLS}}={\left(\Phi^{T}\,\Phi +\lambda \,\text{I}\right)}^{-1}\,\Phi^{T}\,\text{y}$$

where λ is the so-called regularization parameter.

#### Linear parameter varying autoregressive based cross frequency coupling

To quantify the CFC between the phase of a low-frequency rhythm (i.e., phase frequency band *L* with a frequency range of [*L*_*min*_*L*_*max*_]) and the amplitude of a high-frequency rhythm (i.e., amplitude frequency band *H* with a frequency range of [*H*_*min*_*H*_*max*_]), we first apply band-pass filtering in conjunction with the Hilbert transform (Papoulis and Pillai, 2002). Specifically, the raw signal ***x*** ∈ ***R**^N×1^* is band-pass filtered in the low and high frequency bands of interest (i.e., *L* and *H*) using a two-way least-squares finite impulse response filter [Matlab function *eegfilt.m* from EEGLAB toolbox (Delorme and Makeig, 2004)], to preserve phase information. The filter order depends on the low-frequency cut-off value and is defined as the rounded value of three times the ratio of the sampling rate to the low frequency cut-off or else order of three cycles of the lower cut-off frequency. The obtained signals are ***x***_*L*_ ∈ ***R**^N×1^* and ***x***_*H*_ ∈ ***R**^N×1^*. Next, we employ the Hilbert transform to create the analytic signals of ***x***_*L*_ and ***x***_*H*_ and extract the instantaneous phase **P**_*L*_ ∈ ***R**^N×1^* and amplitude **A**_*H*_ ∈ ***R**^N×1^* of the band-pass filtered signals in the frequency bands *L* and *H*, respectively,


(11) $${\text{P}}_{L}=∡\,H\,i\,l\,b\,e\,r\,t\,\left\{{\text{x}}_{L}\right\}\;a\,n\,d\;{\text{A}}_{H}=||H\,i\,l\,b\,e\,r\,t\,\left\{{\text{x}}_{H}\right\}||$$

**FIGURE 1:**
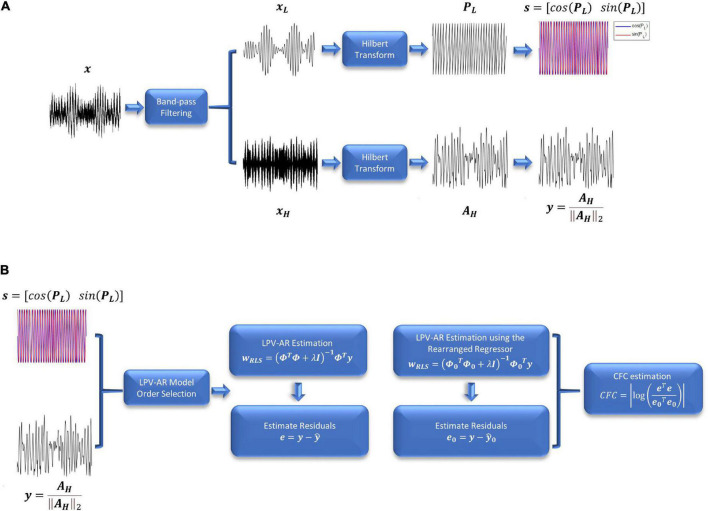
**(A)** Preprocessing steps and **(B)** LPV-AR identification for CFC quantification. **(A)** The signal of interest is first band-pass filtered at the frequency bands *L* and *H*. After applying the Hilbert transform, the instantaneous amplitude and phase signals are used to estimate the LPV-AR based CFC. **(B)** LPV-AR model order selection procedure is realized as described in section “Linear parameter varying autoregressive model order selection.” LPV-AR identification is achieved by estimating the model coefficients. A new LPV-AR model is identified by rearranging the regressor matrix. CFC is quantified based on the residuals of both models.

Note that the Hilbert transform expresses real-valued signals as complex functions with time-varying amplitude and phase (also known as analytic signals). The analytic signals are useful in extracting instantaneous attributes of different time-series. The resulting amplitude and phase envelopes of Eq. 11 are used to assess the phase-amplitude interactions between the low and high frequency bands of interest. To quantify the CFC between the phase of the *L* band and the amplitude of the *H* band, we use the LPV-AR methodology described in section “Linear parameter varying autoregressive model.” The scheduling variable ***s*** is assumed to be the cosine and the sine of the instantaneous phase in the *L* band, whereas the signal of interest ***y*** is the instantaneous amplitude in the *H* band normalized by its norm (to remove any effects from the power of the high frequency envelope) (Figure 1A),


(12) $$\text{y}=\frac{{\text{A}}_{H}}{{∥{\text{A}}_{H}∥}_{2}}\;\mathrm{and}\;\text{s}=\left[\text{cos}\,\left({\text{P}}_{L}\right)\,\text{sin}\,\left({\text{P}}_{L}\right)\right]$$

In Eq. 12 we used the cosine and the sine of the phase of the *L* band to avoid the so-called “null phases” (Penny et al., 2008). Since the scheduling variable ***s*** is now a two-dimensional signal (i.e., ***s*** ∈ ***R**^N×2^* compared to the one-dimensional case in Eq. 2), Eq. 2 becomes,


(13) $$\text{s}=\left[\text{cos}\,\left({\text{P}}_{L}\right)\,\text{sin}\,\left({\text{P}}_{L}\right)\right]=\left[{\text{s}}_{1}\,{\text{s}}_{2}\right]$$

(14) $$y\,\left(n\right)=\sum_{k=1}^{p}a_{k}\,\left({\text{s}}_{n}\right)\,y\,\left(n-k\right)+\varepsilon \,\left(n\right)$$

Based on the polynomial basis, the AR model coefficients {*a*_k_} are now expressed as,


(15) $$a_{k}\left(s_{n}\right)=\theta_{k}^{\text{T}}\Psi_{k}=\theta_{k_{0}}+\sum_{0\leq i+j\leq q}\theta_{k_{i\,j}}s_{1\,n}^{i}s_{2\,n}^{j},k=1:p$$

where for notational simplicity *s*_1*n*_ = *s*_1_(*n*) and *s*_2*n*_ = *s*_2_(*n*). To make models with different polynomial orders more easily identifiable, we retained only the *q*-th order interactions. The final model representation can be expressed as in Eq. 8.

For illustration purposes, assume that *p* = 2, *q* = 2 then Eq. 14 can be written as,


$$y\left(n\right)=\left(\theta_{1_{0}}+\theta_{1_{02}}s_{2\,n}^{2}+\theta_{1_{11}}s_{1\,n}s_{2\,n}+\theta_{1_{20}}s_{1\,n}^{2}\right)y\left(n-1\right)+(\theta_{2_{0}}$$

(16) $$+\theta_{2_{02}}s_{2\,n}^{2}+\theta_{2_{11}}s_{1\,n}s_{2\,n}+\theta_{2_{20}}s_{1\,n}^{2})y(n-2)+\varepsilon (n)$$

To estimate the CFC index between ***s*** and ***y*** we first estimate the coefficients {*a*_*k*_} based on the regularized solution of Eq. 10. The predicted variable of interest $\hat{\text{y}}$ and the residuals **e** of the LPV-AR model are computed as,


(17) $$\hat{\text{y}}=\Phi \,{\text{w}}_{\mathrm{RLS}},\text{e}=\text{y}-\hat{\text{y}}$$

Then, we permute columnwise the order of ***s*** to destroy any dependencies between ***s*** and ***y*** and we recalculate the matrix **Φ** of Eq. 7, generating this way a new regressor matrix **Φ**_0_. The idea behind this procedure is that if the instantaneous phase ***s*** modulates the instantaneous amplitude ***y***, rearranging its values will have a negative impact on the prediction accuracy of the LPV-AR model. On the other hand, if the coupling between the two is not significant, the predictive performance of the model will not be affected strongly. Based on these changes in predictive performance, we can quantify the CFC between ***s*** and ***y***. Using the rearranged regressor matrix **Φ**_0_, the predicted signal of interest and the residuals of the LPV-AR model are given as,


(18) $${\hat{\text{y}}}_{0}=\Phi_{0}\,{\text{w}}_{\mathrm{RLS}},{\text{e}}_{0}=\text{y}-{\hat{\text{y}}}_{0},$$

The CFC index between ***s*** and ***y*** is defined as (Figure 1B),


(19) $$C\,F\,C=\left|\log\left(\frac{{\text{e}}^{T}\,\text{e}}{{\text{e}}_{0}^{T}\,{\text{e}}_{0}}\right)\right|$$

The higher the CFC index, the stronger the coupling between the instantaneous phase and amplitude of the signals of interest.

#### Linear parameter varying autoregressive model order selection

To estimate the CFC index, an optimal model structure should be first selected. The LPV-AR model complexity is essentially defined by the model order *p* and the polynomial order *q*. The higher the *p* and *q*, the better the fit to the observed data but also the lower the predictive performance and thus the generalization capabilities to unseen data. Model order selection revolves around the optimal selection of these two hyperparameters. A common practice is to use cross-validation (CV) or model order selection criteria like the Akaike Information Criterion (Akaike, 1974) or the Bayesian Information Criterion (Schwarz, 1978). For the purposes of CFC estimation, we developed the two-step procedure described below:

1. For a fixed and relatively large regularization parameter λ, we estimate the mean squared error (MSE) on the whole dataset for an ascending order of *p* and *q* values. The *p* and *q* values that achieve the lowest MSE are chosen as optimal.
2. In this step, the *p* and *q* values from step 1 are kept fixed and we optimize the regularization parameter. To determine a suitable value, we use the U-curve technique (Krawczyk-StanDo and Rudnicki, 2007), whereby the sum of the inverse of the norm of the regularized solution and the inverse of the residual norm is plotted in a log-log scale for different regularization parameters (i.e., λ = {10^−3^,10^−2^,10^−1^,1,10,10^2^,10^3^}). The regularization parameter that corresponds to the minimum of the U-curve is then selected as optimal, since it provides a good trade-off between the size of the regularized solution, and its fit to the given data.

The purpose of step 1 is mainly to obtain an appropriate value for the polynomial order *q*, since we found that, based on the simulations and real data described in the following sections, it affects the results. We also observed that all *p* values return a MSE for the same order *q* (see section “Simulations” – Figure 3). The selection of *p* is important but less critical at this step. In step 2, we fine tune the regularization parameter based on the *p* and *q* values acquired at step 1. This way, we make sure that, despite the selection of maybe a fairly complex model in terms of *p* at step 1, a suitable regularization parameter is applied. Apart from controlling model complexity, regularization ensures stability [i.e., poles inside the unit circle (Ljung, 1998)]. Narrowband signals are known to induce temporal instabilities on the AR models, because the roots of the signal generating system are located very close to the unit circle (Hall et al., 1983; Kostoglou and Lunglmayr, 2020). By applying regularization, the chances of obtaining unstable estimates are low. In addition, it provides more consistent model behavior especially when consecutive time samples are highly correlated. This could happen, for example, in lower frequencies that are oversampled (i.e., the bandpass-filtered lower frequency components are not down-sampled. LPV-AR estimation is always conducted in our case using the initial sampling rate).

**FIGURE 2:**
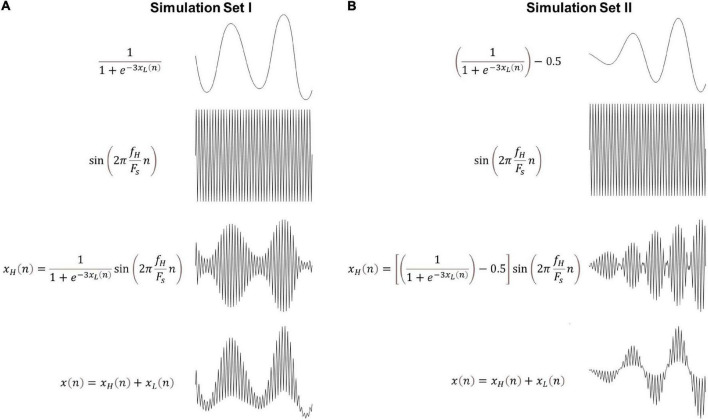
Representative realizations of synthetic signals (Eq. 31) from **(A)** simulation set I (monophasic coupling) and **(B)** simulation set II (biphasic coupling). All depicted signals exhibit CFC between *f*_*L*_ = 4 Hz and *f*_*H*_ = 60 Hz. The sampling rate was set to *F*_*s*_ = 240 Hz and the signal to noise ratio was 40 dB.

**FIGURE 3:**
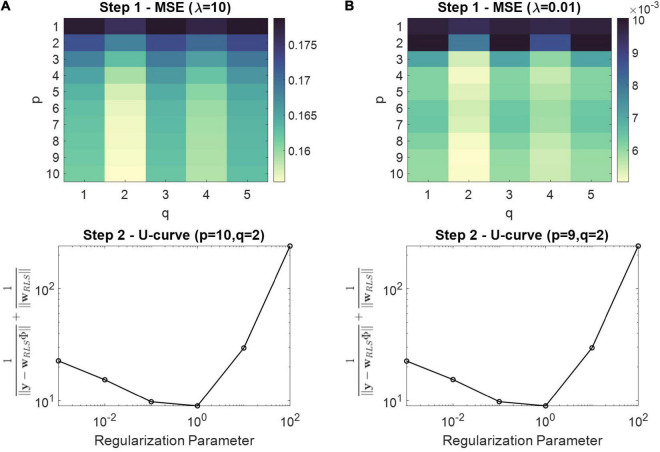
Two step model order selection procedure for one representative realization of Eq. 31 and simulation set I. **(A)** The top panel depicts the MSE obtained during step 1 for different *p* and *q* values using a regularization parameter of λ = 10 as an initial value. The bottom panel depicts the U-curve from step 2, using the model order that achieved the smallest MSE [i.e., (*p*,*q*) = (10,2)] at step 1 (i.e., top panel). The regularization parameter that corresponds to the minimum of the U-curve was selected as optimal (i.e., λ = 1). **(B)** Similar as **(A)**, however, the initial regularization parameter at step 1 was set to λ = 0.01.

### Commonly used cross frequency coupling estimation methods

Here, we provide a brief overview of some of the most used methods for CFC estimation. These methods will be later compared with our proposed LPV-AR approach using both simulated and real EEG data.

#### Mean vector length

To assess CFC Canolty et al. (2006) defined a complex time-series with magnitude equal to the instantaneous high frequency amplitude and angle equal to the instantaneous low frequency phase,


(20) $$\text{z}={\text{A}}_{H}\,e^{j\,P_{L}}$$

The MVL is the absolute value of the mean of *z*,


(21) $$M\,V\,L=||\frac{1}{N}\,\sum_{n=1}^{N}z\,\left(n\right)||$$

In the absence of CFC, vector points of *z*(*n*) in the complex plane exhibit uniform radial symmetry around zero and therefore the MVL takes values close to zero. If there is modulation, then this symmetry is lost and MVL obtains larger values.

#### Phase-locking value

The PLV (Lachaux et al., 1999; Cohen, 2008) is inspired by the well-known phase-locking concept frequently used in neuroscience. For the calculation of the PLV, the high frequency amplitude signal is Hilbert transformed and the phase of the corresponding analytic signal is extracted,


(22) $${\text{P}}_{A_{H}}\,∡\,H\,i\,l\,b\,e\,r\,t\,\left\{{\text{A}}_{H}\right\}$$

PLV is then defined as,


(23) $$P\,L\,V=||\frac{1}{N}\,\sum_{n=1}^{N}e\,x\,p\,\left\{j\,\left[P_{L}\,\left(n\right)-P_{A_{H}}\,\left(n\right)\right]\right\}||$$

When the two phase series ***P**_L_* and ***P**_**A**H_* are locked, then PLV is 1. In case of desynchronization (no CFC) PLV is 0.

#### Kullback–Liebler based modulation index

The Kullback–Liebler based MI by Tort et al. (2010) measures the deviation of the distribution of the high frequency amplitude, with respect to the phase of the low frequency, from the uniform distribution. Specifically, the low frequency phase is binned and the mean of the high frequency amplitude over each phase bin is computed. Each bin value is normalized by the sum over all bins,


(24) $$P\,\left(i\right)=\frac{{<{\text{A}}_{H}>}_{\left(i\right)}}{\sum_{b\,i\,n=1}^{N\,b\,i\,n\,s}{<{\text{A}}_{H}>}_{\left(b\,i\,n\right)}}\;\text{for}\;i=1\,\ldots \,N\,b\,i\,n\,s$$

where < ***A**_H_* > (*i*) refers to the mean high frequency amplitude that corresponds to the *i*-th phase bin. The default value for the number of bins is usually set to *Nbins* = 18. The Shannon entropy of **P** is given as,


(25) $$H\,\left(P\right)=-\sum_{i=1}^{N\,b\,i\,n\,s}P\,\left(i\right)\,\log\left\{P\,\left(i\right)\right\}$$

and the Kullback–Liebler distance (that measures the discrepancy between two distributions) is defined as,


(26) $$D_{K\,L}=\log\left(N\,b\,i\,n\,s\right)-H\,\left(P\right)$$

where log(*Nbins*) is the maximum possible entropy value if the distribution of *P* is uniform. The MI is computed as,


(27) $$M\,I=D_{K\,L}/\log\left(N\,b\,i\,n\,s\right)$$

If the mean amplitude is uniformly distributed over phase (i.e., no CFC), then MI is zero. As the distribution of *P* gets further away from the uniform distribution the Kullback–Liebler distance and the MI increases.

#### Generalized linear model

Penny et al. (2008) introduced the GLM index computed based on a multiple regression model, whereby the high frequency amplitude is expressed a linear combination of the cosine and the sine of the low frequency phase,


(28) $${\text{A}}_{H}=\left[\text{cos}\,\left({\text{P}}_{L}\right)\,\text{sin}\,\left({\text{P}}_{L}\right)\,1\right]\cdot {\text{w}}_{G\,L\,M}+\varepsilon$$

where ε is additive Gaussian noise and *1* is a column of ones. **w**_*GLM*_ can be estimated using the Least Squares solution. The proportion of variance explained by the model is used as the GLM index.

## Simulations

### Signal generation

To validate our approach, we simulated two sets of 100 6 s-long signals that exhibited CFC between *f*_*L*_ = 4 Hz and *f*_*H*_ = 60 Hz at a sampling rate of *F*_*s*_ = 240 Hz. The exact steps followed are summarized below:

1. We bandpass-filtered white Gaussian noise at a center frequency of *f*_*L*_ = 4 Hz and with a bandwidth of 1 Hz using a two-way least squares FIR filter as described in section “Linear parameter varying autoregressive based cross frequency coupling.” The obtained signal ***x***_*L*_ was then normalized to unit variance.
2. The amplitude of a sinusoid at *f*_*H*_ = 60 Hz was modulated by the signal ***x***_*L*_ (of step 1) as follows (Figure 2),

(29) $$\mathrm{Simulation}\mathrm{set}I:\;x_{H}\left(n\right)=\frac{1}{1+e^{-3\,x_{L}\,\left(n\right)}}\sin\left(2\pi \frac{f_{H}}{F_{s}}n\right)$$

$$\mathrm{Simulation}\mathrm{set}\mathrm{II}:\;x_{H}\left(n\right)=\left(\frac{1}{1+e^{-3\,x_{L}\,\left(n\right)}}-0.5\right)$$

(30) $$\sin\left(2\,\pi \,\frac{f_{H}}{F_{s}}\,n\right)$$

3. The final signal was computed as,

(31) $$x\,\left(n\right)=x_{H}\,\left(n\right)+x_{L}\,\left(n\right)+u\,\left(n\right)$$

where ***u*** is zero-mean white Gaussian noise with standard deviation σ_*u*_ = 0.5 (around 40 dB signal to noise ratio).

In the two simulation sets the amplitude of the high frequency is modulated differently by the phase of the low frequency. Specifically, in set I, the high frequency amplitude is maximum during the peaks of the low frequency, known as monophasic coupling, whereas in set II, it attains its maximum during the peaks but also the troughs of the low frequency component, known as biphasic coupling. For each simulation set, we generated different realizations of Eq. 31 by repeating steps 1–3 100 times (i.e., ***x***_*L*_ and ***u*** were produced based on different Gaussian white noise realizations). Some representative examples for both simulation sets can be found in Figure 2.

### Quantifying cross frequency coupling using comodulograms

Comodulograms are coupling maps that depict the CFC strength as a function of the phase frequency and the amplitude frequency. For each realization of simulation sets I and II, we created comodulograms for amplitude frequencies ranging between 20 and 80 Hz (with a 2 Hz step and a bandwidth of 2 Hz, i.e., 20–22, 22–24 Hz, etc.) and phase frequencies between 2 and 10 Hz (with a 1 Hz step and a bandwidth of 0.5 Hz). LPV-AR based CFC was estimated at each amplitude and phase frequency band as described in sections “Linear parameter varying autoregressive based cross frequency coupling” and “Linear parameter varying autoregressive model order selection.” Model order selection was realized at each bin. To compare our approach with other methods, we analyzed the average comodulograms, over all 100 realizations, extracted in each case.

## EEG data

### Experimental protocol and preprocessing

In total, 61-channel EEG signals were obtained from 8, originally right-handed, SCI participants (median ± SD age of 54 ± 18) with a neurological level of injury ranging from C1 to C7 as described in Ofner et al. (2019).^1^ A fixation cross along with a beep sound was presented on a black screen to signal the beginning of the trial. Class cues were displayed 2 s after the trial initialization and each trial lasted for 5 s. The participants were asked to attempt unilaterally arm/hand movements such as hand open, supination, pronation, palmar grasp and lateral grasp. Each attempted movement class consisted of 72 trials.

The recorded EEG signals were pre-processed using EEGLAB and Matlab. Bandpass filtering between 0.3 and 70 Hz was applied to the signals using a fourth order, zero-phase, Butterworth filter. Trials with impulsive noise were rejected using thresholding. Techniques based on abnormal joint probabilities and kurtosis (Ofner et al., 2019) were also employed in order to eliminate noisy trials. Blinks, saccades, and muscle movements were removed using independent component analysis.

To reduce the dimensionality of the data we defined the following nine regions of interest (ROI) in the sensor space: **F_*Z*_** (average of channels FCz and Fz), **F_*L*_** (average of channels F3, F1, FFC5h, FFC3h, FFC1h, FC5, FC3, FC1, FCC5h, FCC3h, and FCC1h), **F_*R*_** (average of channels F2, F4, FFC2h, FFC4h, FFC5h, FC2, FC4, FC6, FCC2h, FCC4h, and FCC6h), **C_*Z*_** (average of channels Cz and CPz), **C_*L*_** (average of channels C5, C3, C1, CCP5h, CCP3h, CCP1h, CP5, CP3, CP1, CPP5h, CPP3h, and CPP1h), **C_*R*_** (average of channels C2, C4, C6, CCP2h, CCP4h, CCP6h, CP2, CP4, CP6, CPP2h, CPP4h, and CPP6h), **P_*Z*_** (average of channels Pz and POz), **P_*L*_** (average of channels P5, P3, P1, and PPO1h), and **P_*R*_** (average of channels P2, P4, P6, and PPO2h). Note that in order to estimate CFC we first filtered the signals in the frequency bands of interest and then we averaged over different ROIs.

### Cross frequency coupling estimation

For each subject and each attempted movement, we estimated CFC on a ROI-by-ROI and trial-by-trial basis and in the following frequency bands of interest: *delta* (D: [0.5 3] Hz), *theta* (T: [4 8]), *alpha* (A: [8.5 12]), *beta* (B: [12.5 30]), *gamma* (G: [30.5 60]), and *high gamma* (HG: [60 70]). The pair of phase and amplitude frequency bands [*L*,*H*] (i.e., *CFC*_*LH*_) were [*D*,*T*],[*D*,*A*],[*D*,*B*], [*D*,*G*],[*D*,*HG*],[*T*,*A*],[*T*,*B*],[*T*,*G*],[*T*,*HG*],[*A*,*B*], [*A*,*G*],[*A*,*HG*],[*B*,*G*],[*B*,*HG*],[*G*,*HG*]. Instead of using the whole range of the phase frequency band, we defined as the low frequency phase driver the sub-band with the highest power (by bandpass-filtering the signals in incremental steps of 0.5 and 1 Hz bandwidth and estimating their corresponding power). Therefore, for each trial we extracted 135 CFC indices (15 indices × 9 ROI). Trial-based CFC was computed using the same techniques investigated in the simulations section, along with the LPV-AR approach. LPV-AR model order selection and estimation was conducted for each trial and for each pair of low and high frequency bands. The extracted CFC indices were then used as features for movement attempt classification. We considered only data segments corresponding to the period between the cue onset and the end of the trial (3 s long).

### Movement attempt classification using cross frequency coupling

We classified movement attempts using a multiclass shrinkage linear discriminant analysis classifier (Peck and Van Ness, 1982; Ofner et al., 2019). We focused on five types of movements: hand open, pronation, supination, palmar grasp and lateral grasp. We trained different classifiers for each subject and for each CFC estimation method. Trial-based classification performance was assessed using accuracy.

To reduce the number of features and improve classification performance, for each method and each subject, we applied a backward elimination feature selection scheme. Initially we trained a classifier with 135 CFC indices. To assess feature importance, we randomly shuffled the values of each feature in the testing sets and computed the average change in accuracy. The idea is that if a feature is important, rearranging its values will lead to a drop in accuracy performance. If the feature, however, is non-informative then the testing set accuracy will not be affected significantly. At each iteration, the feature with the lowest importance was removed and new classifiers were trained. This procedure was repeated until no features were left. Based on this method, the accuracy increased with the number of features, reached a maximum point, and then decreased again. The number of features that corresponded to this maximum point, was selected as optimal.

To validate the classification results and the feature selection procedure, we used both external and internal CV schemes. The external CV was used to quantify the generalization capabilities of the classifier on unseen data (i.e., 10% hold-out). The internal CV (fivefold) was used for feature selection, i.e., different classifiers were trained on each training set and feature importance was assessed based on the mean CV testing performance. To test for statistical significance and eliminate the hypothesis of possible overfitting, the class labels were permuted randomly and the whole feature selection and classification procedure was repeated using the same train/test/validation sets applied on the non-permuted data. To assess the overall performance in different hold out sets during the external CV, the whole internal/external CV scheme was employed 50 times by randomly resampling 10% of the data for hold-out. To predict the hold-out set, for each subset of features, the classifier was retrained based on all the data used for internal CV.

### Comparison of different cross frequency coupling estimation methods based on the classification results

Our main assumption was that the CFC method that produced the highest classification performance would also provide more accurate CFC estimates. For a fair comparison, we investigated classification performances based on different number of features. This is also one of the reasons that we applied the feature selection procedure of section “Movement attempt classification using cross frequency coupling” for each CFC estimation method.

## Results

### Simulations

In Figure 3 we illustrate the results obtained from the two-step model order selection procedure for a representative realization of simulation set I. The top panels depict the MSE obtained during step 1 for different *p* and *q* values using a fixed regularization parameter (i.e., λ = 10 and λ = 0.01 for Figures 3A,B, respectively). The bottom panels depict the U-curves extracted during step 2, using the model order that achieved the smallest MSE at step 1 (i.e., top panels). The regularization parameter that corresponds to the minimum of the U-curve was selected as optimal. The difference between Figures 3A,B is the value of the regularization parameter that was used for step 1. In both cases, however, we ended up with the same polynomial order of *q* = 2, which produces the smallest MSE for all *p* values. Therefore, step 1 is important in terms of selecting and appropriate *q* value, and step 2 is applied to regularize the complexity imposed by the selected *p* from step 1.

**FIGURE 4:**
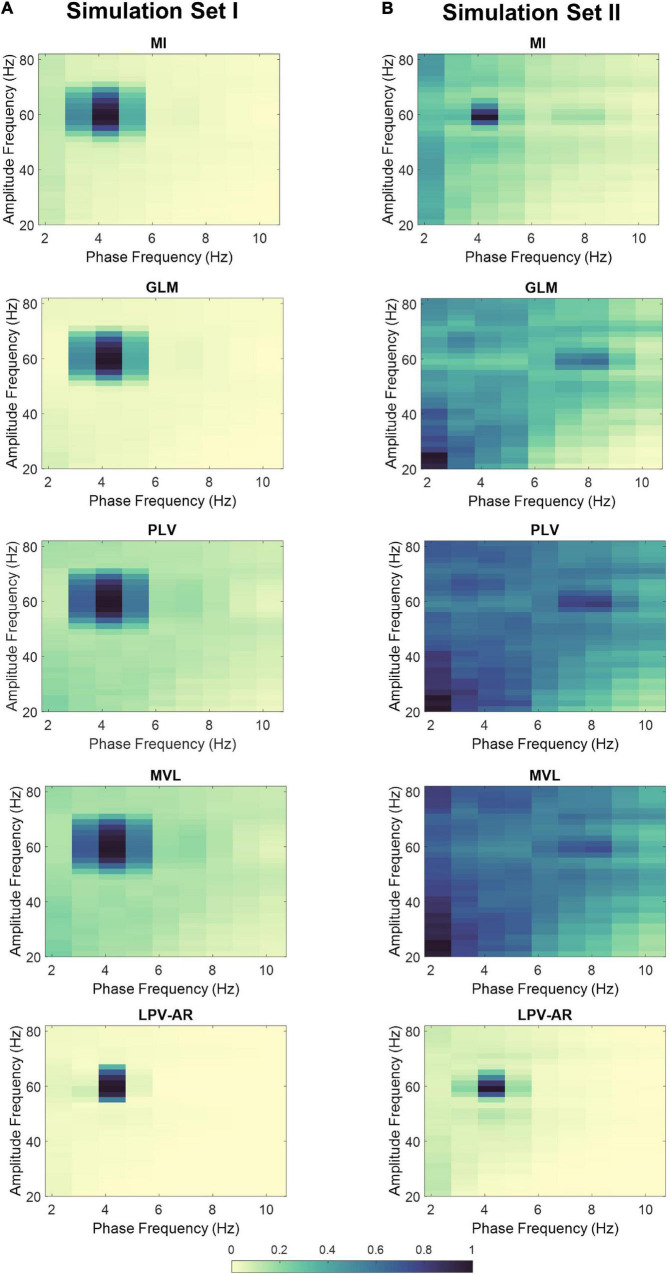
Average comodulograms over 100 realizations for **(A)** simulation set I and **(B)** simulation set II. Each row corresponds to a different method (i.e., MI first row, GLM second row, PLV third row, MVL fourth row, and LPV-AR fifth row). For comparison purposes, the average comodulogram of each method was normalized by its maximum value over all phase and amplitude bins.

**FIGURE 5:**
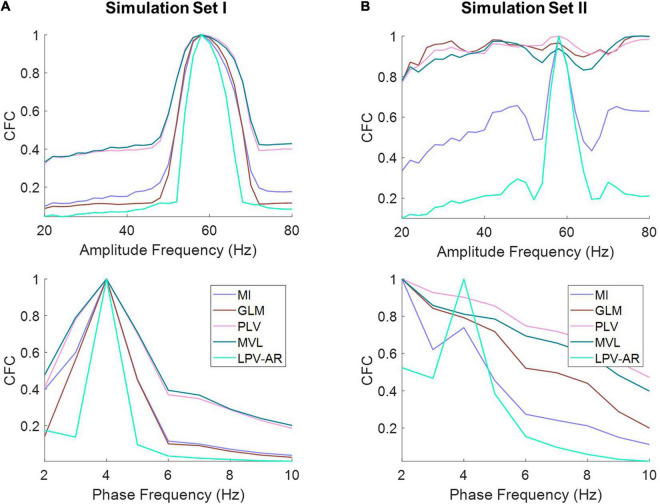
The average of the comodulograms depicted in Figure 4 for **(A)** simulation set I and **(B)** simulation set II along the amplitude (top panels) and the phase frequencies (bottom panel) for different CFC methods (normalized to their maximum value). The LPV-AR technique exhibits higher resolution in detecting CFC (i.e., higher CFC at *f*_*L*_ = 4 Hz and *f*_*H*_ = 60 Hz and lower sidebands around these frequencies) and lower bias in frequencies where no coupling exists.

In Figure 4 we illustrate the average comodulograms obtained over all 100 realizations for both simulation sets and for different CFC estimation methods. The 2D comodulograms were averaged either over all phase frequencies or over all amplitude frequencies producing the results illustrated in Figure 5. For the simulation set I (i.e., the monophasic case), all methods were able to detect CFC between *f*_*L*_ = 4 Hz and *f*_*H*_ = 60 Hz. However, the LPV-AR methodology exhibited higher resolution detection (i.e., higher CFC at *f*_*L*_ = 4 Hz and *f*_*H*_ = 60 Hz and lower sidebands around these frequencies) and lower bias in frequencies where no coupling existed. For the simulation set II, the biphasic nature of the coupling led to distorted measures of CFC in most algorithms. The LPV-AR methodology achieved the best CFC detection results, followed by the MI technique. The GLM, PLV, and MVL were not able to detect the coupled phase and amplitude frequencies correctly. In terms of computational complexity, the LPV-AR model requires a larger number of operations and therefore its runtime is expected to be higher. The mean runtime was 0.26 ± 0.06 ms for MI, 0.31 ± 1.70 ms for GLM, 1.13 ± 0.48 ms for PLV, 0.06 ± 0.01 ms for MVL, and 84.52 ± 14.88 ms for LPV-AR.

### EEG data

**FIGURE 6:**
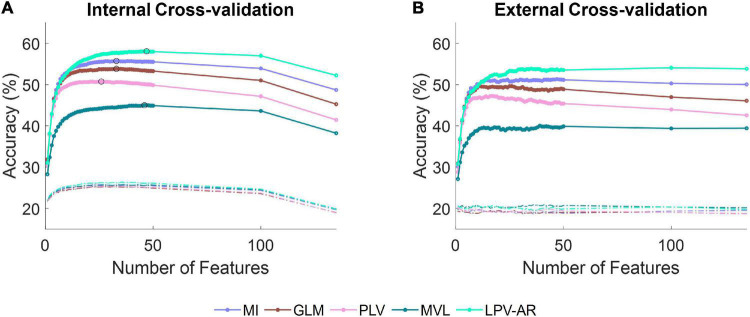
Average accuracy over all participants for the five-class classification problem (i.e., hand open vs. palmar grasp vs. lateral grasp vs. pronation vs. supination) as a function of the number of features during **(A)** the internal cross-validation and **(B)** the external cross-validation step. Different line colors correspond to different CFC estimation methods. Dashed lines represent accuracy levels obtained by training the classifier on data with permuted labels. The theoretical chance accuracy level is 20%. In **(A)**, the maximum classification accuracy for each CFC method is indicated with a black circle.

The average accuracy plots (over all participants and over all hold out sets) for the five-class EEG classification problem (i.e., hand open vs. palmar grasp vs. lateral grasp vs. pronation vs. supination) as a function of the number of features can be found in Figure 6. The LPV-AR approach achieved the highest accuracy (Internal CV: 58 ± 5% – External CV: 54 ± 5%), followed by the MI (Internal CV: 55 ± 5% – External CV: 51 ± 5%), the GLM (Internal CV: 53 ± 4% – External CV: 49 ± 5%), the PLV (Internal CV: 50 ± 5% – External CV: 47 ± 6%), and finally the MVL (Internal CV: 45 ± 3% – External CV: 40 ± 3%). Based on the Kruskal–Wallis test, for 47 features (number of features that correspond to the maximum internal CV accuracy for the LPV-AR model) significant differences in accuracy performance were detected between methods. By applying the Dunn’s *post hoc* test for multiple comparisons analysis, the LPV-AR model was found to perform significantly better than the other methods in both external and internal CV (*p* < 0.05).

**FIGURE 7:**
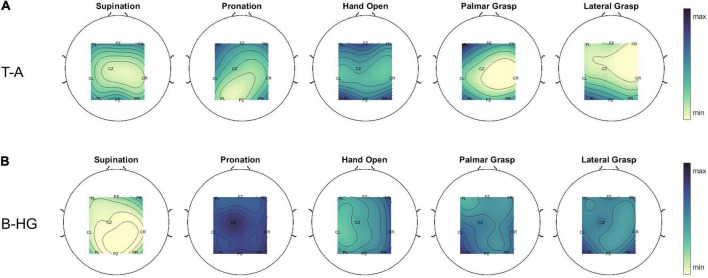
Average topoplots (over all participants and trials) of the estimated LPV-AR CFC between **(A)** the phase of the *theta* band and the amplitude of the *alpha* band and **(B)** the phase of the *beta* band and the amplitude of the *high gamma* band for the five different movement attempts. In **(A,B)**, max and min refers to the maximum and minimum value, respectively, obtained from all five movement types.

**FIGURE 8:**
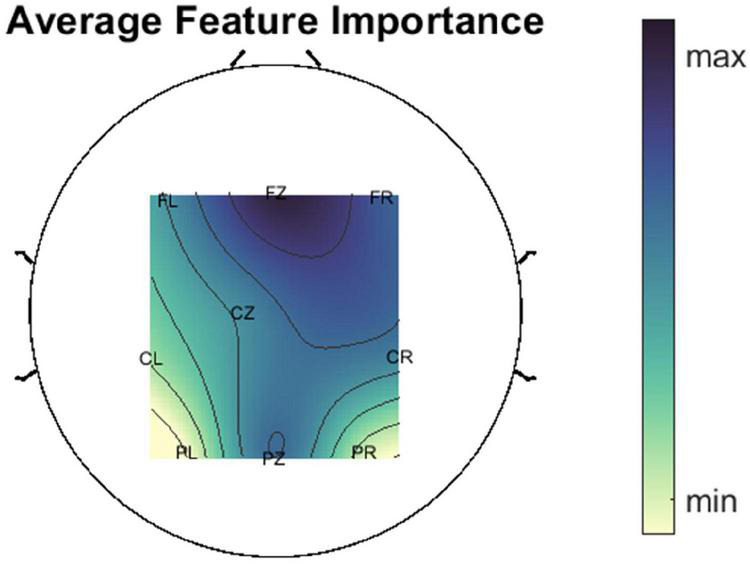
Average ROI importance. The importance of CFC features belonging to a specific ROI for all low and high frequency band combinations were averaged providing this way a measure of ROI importance.

**FIGURE 9:**
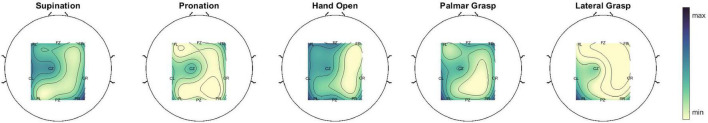
Overall effect of the *delta* band on all frequency bands. The topoplots were averaged over all participants and trials for the five movement attempts. They represent the average CFC between the *delta* band and all other frequency bands.

Cross frequency coupling features were ranked by averaging their importance over all subjects and over all 50 internal/external CV repetitions. Some of the top features were the CFC between the *theta* and *alpha* in the **F_*Z*_** region, the CFC between the *beta* and *high gamma* in the **F_*L*_** region, the CFC between the *delta* and *alpha* in the **F_*Z*_** region and the CFC between the *theta* and *alpha* in the **C_*Z*_** region. Some representative topoplots of LPV-AR CFC for the five different attempted movements can be found in Figure 7. In Figure 8 we present the average importance of different ROIs (i.e., the importance of CFC features belonging to a specific ROI, for all low and high frequency band combinations, were averaged creating this way Figure 8). The ROIs ranked based on importance from highest to lowest were: **F_*Z*_**, **F_*R*,_ P_*Z*,_ C_*R*,_ C_*Z*,_ F_*L*,_ C_*L*,_ P_*R*,_ P_*L*_**. Ipsilateral frontal and fronto-central areas exhibited higher discriminative power, followed by the parietal and the central regions. Overall, the highest CFC was induced by the *delta* band as phase frequency band. The effect of the *delta* on all frequency bands can be seen in Figure 9.

## Discussion

In the present study, we developed a CFC estimation technique based on LPV-AR models. Specifically, we allowed the phase of the lower frequency rhythms to modulate the coefficients of an AR representation of the higher frequency oscillations. The effect of the low frequency phase on the high frequency amplitude was quantified based on the residuals of the model and used as an index of CFC.

la Tour et al. (2017), have previously suggested the use of LPV-AR models (referred to as driven AR models) for CFC estimation. Their technique relied on removing the low frequency component (i.e., ***x***_*L*_ in our case) from the raw signal. The frequency gap in the obtained time series was then filled by adding filtered gaussian white noise based on the filter that removed initially the corresponding low frequency driver. This signal was then used as the ***y*** variable in Eq. 14 and ***x***_*L*_ was defined as the scheduling variable. The main motivation behind this procedure was the use of a persistently exciting signal for AR fitting, since AR modeling provides more robust and stable estimates on a broad spectral structure (Hall et al., 1983; Kostoglou and Lunglmayr, 2020). After model estimation, the conditional power spectral density of the model, which essentially describes the power changes of ***y*** in different frequencies with respect to the scheduling variable, was used in conjunction with the Kullback–Leibler divergence metric and the low frequency phase to quantify the CFC (similarly as in the MI method). Here, we followed a computationally more efficient approach, whereby the narrowband high frequency envelope and the low frequency phase was fed directly to the LPV-AR model. Stability issues were dealt with by applying regularization on the model coefficients and CFC was quantified by computing the relative change in the norm of the model residuals when the phase values were permuted randomly. Possible effects of the power of the high frequency amplitude on the CFC indices were mitigated by normalizing the envelope signal by its norm prior to the LPV-AR estimation. It would be interesting in the future to compare these two techniques.

Compared to other well-established CFC methods, our proposed LPV-AR approach exhibits some rather important advantages. Based on synthetic data, the LPV-AR method displayed lower CFC bias in amplitude and phase frequencies that were not coupled, and its detection resolution was higher compared to other methods. An important factor that affected the CFC estimation results was the multimodality of the coupling. Biphasic coupling led to poorer performance in most methods apart from the LPV-AR (followed by the MI) which was able to accurately detect CFC at the correct phase and amplitude frequencies. This is mainly due to the mathematic construct of most methods that prevents the detection of multimodal CFC. The main disadvantage of the LPV-AR approach was its computational complexity. The LPV-AR runtime was higher compared to the other algorithms. However, improvements could be made to speed up computations, i.e., the coding could be made more efficient or parallel processing could be applied.

An important advantage of the LPV-AR is the possibility of extracting various model-based features that could be used either for classification or for a deeper understanding of the underlying couplings and dynamics. For example, based on the model coefficients one may estimate the time-varying power spectrum of the signal under consideration as a function of the external scheduling variable. Most importantly, however, is that time-varying CFC can be estimated directly using Eq. 19. Instead of taking the norm of the errors, the instantaneous squared errors can be used to obtain the time evolution of the CFC index. This can provide a more accurate time-varying representation compared to other methods that require the use of overlapping sliding windows the length of which must be defined and tested. Future works revolves around investigating the time-varying performance of the LPV-AR models and providing more concrete results.

In the simulation sections, we have not covered thoroughly all possible factors and conditions that could affect CFC estimation as in (Penny et al., 2008; Tort et al., 2010; la Tour et al., 2017; Hülsemann et al., 2019; Jurkiewicz et al., 2021). We rather focused on the unimodality or the multimodality of the coupling. Nonetheless, we opted to use real EEG recordings and compare the different algorithms under realistic scenarios. This was done indirectly by comparing classification accuracies during SCI movement attempts using features derived from the extracted CFC estimates of each method. Our main assumption was that if an algorithm produces more accurate CFC estimates, this will be projected onto the classification accuracies. And indeed, our results support this assumption. The LPV-AR technique achieved the highest accuracy compared to the four other commonly used CFC estimation methods.

A methodological aspect that had a significant impact in performance was assigning as low frequency phase driver the sub-band with the highest power (instead of using the whole low frequency band to extract the instantaneous phase signal). This was mainly inspired by Aru et al. (2015) who suggested that meaningful CFC interpretation relies on the existence of a clear peak in the power spectrum of the low frequency component. We believe that this approach provides less noisy CFC estimates and allows for robust detection of CFC patterns in the data. Furthermore, to take advantage of the high spectral CFC resolution detection of the LPV-AR technique, the selection of a narrow sub-band as the main low frequency driver was deemed more appropriate than using the whole phase frequency band.

Although our goal was not to achieve maximum classification performance, the results corroborate the potentiality of CFC as a feature in future BCI applications. The accuracies acquired here are similar and slightly better to the ones obtained by Ofner et al. (2019) on the same dataset, indicating that CFC can also carry important information. Possibly a combination of time-domain features as used in Ofner et al. (2019) and CFC indices could lead to even higher classification performances.

Here, we have provided regions and frequency bands that play an important role in differentiating the investigated attempted movements. Ipsilateral and frontal/frontocentral regions exhibited in general higher discriminative power. We observed that overall, the *delta* band was the phase frequency that induced the largest CFC. This was expected since Movement Related Cortical Potentials (MR) reside in that frequency band and are generated during attempted movements (Ofner et al., 2019). The maximum overall effect of the *delta* band was detected mainly in central and contralateral to the attempted movement areas (i.e., left hemisphere), which was expected since MRCPs have been observed in central and lateral motor areas (Pereira et al., 2017; Ofner et al., 2019; Schwarz et al., 2020). The interpretation of the results is not the main focus of this work but in the future, it will be interesting to incorporate such an aspect into our analysis.

## Conclusion

We present an alternative method for estimating CFC based on LPV-AR models. The LPV-AR approach assumes that high frequency oscillations are AR processes with time-varying coefficients driven by the phase of the lower frequencies. We provide a complete framework with all necessary steps for model order selection/estimation and CFC quantification. Our findings using both simulations and EEG data support our approach and pave the way toward using CFC as a decoding feature in motor related BCI applications. Our work can also be extended, in the future, in finding common CFC mechanisms among healthy and SCI participants under different types of movement modalities.

## Data availability statement

The original contributions presented in this study are included in the article/supplementary material, further inquiries can be directed to the corresponding author.

## Author contributions

KK conducted the analysis and wrote the initial draft. KK and GM-P interpreted the data, performed the proofreading, and finalized the manuscript. Both authors contributed to the article and approved the submitted version.

## Footnotes

1 http://bnci-horizon-2020.eu/database/data-sets, Dataset No. 26.

## References

[B1] Akaike H. (1974). A new look at the statistical model identification. *IEEE Trans. Autom. Control* 19 716–723. 10.1109/TAC.1974.1100705

[B2] Aru J. Aru J. Priesemann V. Wibral M. Lana L. Pipa G. (2015). Untangling cross-frequency coupling in neuroscience. *Curr. Opin. Neurobiol.* 31 51–61. 25212583 10.1016/j.conb.2014.08.002

[B3] Axmacher N. Henseler M. M. Jensen O. Weinreich I. Elger C. E. Fell J. (2010). Cross-frequency coupling supports multi-item working memory in the human hippocampus. *Proc. Natl. Acad. Sci. U.S.A.* 107 3228–3233. 10.1073/pnas.0911531107 20133762 PMC2840289

[B4] Canolty R. T. Edwards E. Dalal S. S. Soltani M. Nagarajan S. S. Kirsch H. E. (2006). High gamma power is phase-locked to theta oscillations in human neocortex. *Science* 313 1626–1628. 10.1126/science.1128115 16973878 PMC2628289

[B5] Canolty R. T. Knight R. T. (2010). The functional role of cross-frequency coupling. *Trends Cogn. Sci.* 14 506–515. 10.1016/j.tics.2010.09.001 20932795 PMC3359652

[B6] Cohen M. X. (2008). Assessing transient cross-frequency coupling in EEG data. *J. Neurosci. Methods* 168 494–499. 10.1016/j.jneumeth.2007.10.012 18061683

[B7] Cohen M. X. Elger C. E. Fell J. (2008). Oscillatory activity and phase–amplitude coupling in the human medial frontal cortex during decision making. *J. Cogn. Neurosci.* 21 390–402. 10.1162/jocn.2008.21020 18510444

[B8] Combrisson E. Perrone-Bertolotti M. Soto J. L. P. Alamian G. Kahane P. Lachaux J.-P. (2017). From intentions to actions: Neural oscillations encode motor processes through phase, amplitude and phase-amplitude coupling. *Neuroimage* 147 473–487. 10.1016/j.neuroimage.2016.11.042 27915117

[B9] De Hemptinne C. Ryapolova-Webb E. S. Air E. L. Garcia P. A. Miller K. J. Ojemann J. G. (2013). Exaggerated phase–amplitude coupling in the primary motor cortex in Parkinson disease. *Proc. Natl. Acad. Sci. U.S.A.* 110 4780–4785. 10.1073/pnas.1214546110 23471992 PMC3606991

[B10] De Lange F. P. Jensen O. Bauer M. Toni I. (2008). Interactions between posterior gamma and frontal alpha/beta oscillations during imagined actions. *Front. Hum. Neurosci.* 2:7. 10.3389/neuro.09.007 PMC2572199 18958208

[B11] Delorme A. Makeig S. (2004). EEGLAB: An open source toolbox for analysis of single-trial EEG dynamics including independent component analysis. *J. Neurosci. Methods* 134 9–21. 10.1016/j.jneumeth.2003.10.009 15102499

[B12] Dimitriadis S. I. Marimpis A. D. (2018). Enhancing performance and bit rates in a brain–computer interface system with phase-to-amplitude cross-frequency coupling: Evidences from traditional c-VEP, Fast c-VEP, and SSVEP designs. *Front. Neuroinform.* 12:19. 10.3389/fninf.2018.00019 29867425 PMC5952007

[B13] Edakawa K. Yanagisawa T. Kishima H. Fukuma R. Oshino S. Khoo H. M. (2016). Detection of epileptic seizures using phase–amplitude coupling in intracranial electroencephalography. *Sci. Rep.* 6:25422. 10.1038/srep25422 27147119 PMC4857088

[B14] Feng N. Hu F. Wang H. Gouda M. A. (2020). Decoding of voluntary and involuntary upper-limb motor imagery based on graph fourier transform and cross-frequency coupling coefficients. *J. Neural Eng.* 17:056043. 10.1088/1741-2552/abc024 33045685

[B15] Georgiadis K. Laskaris N. Nikolopoulos S. Kompatsiaris I. (2019). Connectivity steered graph Fourier transform for motor imagery BCI decoding. *J. Neural Eng.* 16:56021. 10.1088/1741-2552/ab21fd 31096192

[B16] Gwon D. Ahn M. (2021). Alpha and high gamma phase amplitude coupling during motor imagery and weighted cross-frequency coupling to extract discriminative cross-frequency patterns. *Neuroimage* 240:118403. 10.1016/j.neuroimage.2021.118403 34280525

[B17] Gysels E. Celka P. (2004). Phase synchronization for the recognition of mental tasks in a brain-computer interface. *IEEE Trans. Neural Syst. Rehabil. Eng.* 12 406–415. 10.1109/TNSRE.2004.838443 15614996

[B18] Hall M. G. Oppenheim A. V. Willsky A. S. (1983). Time-varying parametric modeling of speech. *Signal Process.* 5 267–285. 10.1016/0165-1684(83)90074-9

[B19] Hülsemann M. J. Naumann E. Rasch B. (2019). Quantification of phase-amplitude coupling in neuronal oscillations: Comparison of phase-locking value, mean vector length, modulation index, and generalized-linear-modeling-cross-frequency-coupling. *Front. Neurosci.* 13:573. 10.3389/fnins.2019.00573 31275096 PMC6592221

[B20] Jurkiewicz G. J. Hunt M. J. Żygierewicz J. (2021). Addressing pitfalls in phase-amplitude coupling analysis with an extended modulation index toolbox. *Neuroinformatics* 19 319–345. 10.1007/s12021-020-09487-3 32845497 PMC8004528

[B21] Kostoglou K. Lunglmayr M. (2020). Root tracking using time-varying autoregressive moving average models and sigma-point Kalman filters. *EURASIP J. Adv. Signal Process.* 2020:6. 10.1186/s13634-020-00666-7

[B22] Krawczyk-StanDo D. Rudnicki M. (2007). Regularization parameter selection in discrete ill-posed problems–The use of the U-Curve. *Int. J. Appl. Math. Comput. Sci.* 17 157–164. 10.2478/v10006-007-0014-3

[B23] la Tour T. Tallot L. Grabot L. Doyère V. Van Wassenhove V. Grenier Y. (2017). Non-linear auto-regressive models for cross-frequency coupling in neural time series. *PLoS Comput. Biol.* 13:e1005893. 10.1371/journal.pcbi.1005893 29227989 PMC5739510

[B24] Lachaux J.-P. Rodriguez E. Martinerie J. Varela F. J. (1999). Measuring phase synchrony in brain signals. *Hum. Brain Mapp.* 8 194–208. 10619414 10.1002/(SICI)1097-0193(1999)8:4<194::AID-HBM4>3.0.CO;2-C PMC6873296

[B25] Ljung L. (1998). *System identification.* Berlin: Springer.

[B26] López-Azcárate J. Tainta M. Rodríguez-Oroz M. C. Valencia M. González R. Guridi J. (2010). Coupling between beta and high-frequency activity in the human subthalamic nucleus may be a pathophysiological mechanism in Parkinson’s disease. *J. Neurosci.* 30 6667–6677. 10.1523/JNEUROSCI.5459-09.2010 20463229 PMC6632566

[B27] Marmarelis V. Z. (2004). *Nonlinear dynamic modeling of physiological systems.* Hoboken, NJ: John Wiley & Sons.

[B28] Mitsis G. D. Marmarelis V. Z. (2002). Modeling of nonlinear physiological systems with fast and slow dynamics. I. Methodology. *Ann. Biomed. Eng.* 30 272–281. 11962778 10.1114/1.1458591

[B29] Mormann F. Fell J. Axmacher N. Weber B. Lehnertz K. Elger C. E. (2005). Phase/amplitude reset and theta–gamma interaction in the human medial temporal lobe during a continuous word recognition memory task. *Hippocampus* 15 890–900. 10.1002/hipo.20117 16114010

[B30] Ofner P. Schwarz A. Pereira J. Wyss D. Wildburger R. Müller-Putz G. R. (2019). Attempted arm and hand movements can be decoded from low-frequency EEG from persons with spinal cord injury. *Sci. Rep.* 9:7134. 10.1038/s41598-019-43594-9 31073142 PMC6509331

[B31] Özkurt T. E. Schnitzler A. (2011). A critical note on the definition of phase–amplitude cross-frequency coupling. *J. Neurosci. Methods* 201 438–443. 10.1016/j.jneumeth.2011.08.014 21871489

[B32] Papoulis A. Pillai S. U. (2002). *Probability, random variables, and stochastic processes.* New York, NY: McGraw-Hill Education.

[B33] Peck R. Van Ness J. (1982). The use of shrinkage estimators in linear discriminant analysis. *IEEE Trans. Pattern Anal. Mach. Intell.* 4 530–537. 10.1109/TPAMI.1982.4767298 21869073

[B34] Penny W. D. Duzel E. Miller K. J. Ojemann J. G. (2008). Testing for nested oscillation. *J. Neurosci. Methods* 174 50–61. 10.1016/j.jneumeth.2008.06.035 18674562 PMC2675174

[B35] Pereira J. Ofner P. Schwarz A. Sburlea A. I. Müller-Putz G. R. (2017). EEG neural correlates of goal-directed movement intention. *Neuroimage* 149 129–140. 10.1016/j.neuroimage.2017.01.030 28131888 PMC5387183

[B36] Schwarz A. Höller M. K. Pereira J. Ofner P. Müller-Putz G. R. (2020). Decoding hand movements from human EEG to control a robotic arm in a simulation environment. *J. Neural Eng.* 17:36010. 10.1088/1741-2552/ab882e 32272464

[B37] Schwarz G. (1978). Estimating the dimension of a model. *Ann. Stat.* 6 461–464. 10.1007/978-3-319-10470-6_18 25485372

[B38] Seeber M. Scherer R. Wagner J. Solis-Escalante T. Müller-Putz G. R. (2014). EEG beta suppression and low gamma modulation are different elements of human upright walking. *Front. Hum. Neurosci.* 8:485. 10.3389/fnhum.2014.00485 25071515 PMC4086296

[B39] Tort A. B. L. Komorowski R. Eichenbaum H. Kopell N. (2010). Measuring phase-amplitude coupling between neuronal oscillations of different frequencies. *J. Neurophysiol.* 104 1195–1210. 10.1152/jn.00106.2010 20463205 PMC2941206

[B40] Tort A. B. L. Komorowski R. W. Manns J. R. Kopell N. J. Eichenbaum H. (2009). Theta–gamma coupling increases during the learning of item–context associations. *Proc. Natl. Acad. Sci. U.S.A.* 106 20942–20947. 10.1073/pnas.0911331106 19934062 PMC2791641

[B41] Tóth R. (2010). *Modeling and identification of linear parameter-varying systems.* Berlin: Springer.

[B42] Wang J. Fang Y. Wang X. Yang H. Yu X. Wang H. (2017). Enhanced gamma activity and cross-frequency interaction of resting-state electroencephalographic oscillations in patients with Alzheimer’s disease. *Front. Aging Neurosci.* 9:243. 10.3389/fnagi.2017.00243 28798683 PMC5526997

[B43] Wei Q. Wang Y. Gao X. Gao S. (2007). Amplitude and phase coupling measures for feature extraction in an EEG-based brain–computer interface. *J. Neural Eng.* 4 120–129. 10.1088/1741-2560/4/2/012 17409486

[B44] Zhang Z. G. Hung Y. S. Chan S.-C. (2010). Local polynomial modeling of time-varying autoregressive models with application to time–frequency analysis of event-related EEG. *IEEE Trans. Biomed. Eng.* 58 557–566. 10.1109/TBME.2010.2089686 20977980

